# Bouveret Syndrome: A Rare Cause of Gastric Outlet Obstruction and Treatment Options

**DOI:** 10.7759/cureus.77032

**Published:** 2025-01-06

**Authors:** Aqsa Khan, Tooba Khan, Kamran Mushtaq, Christina Zelt, Neil Sharma

**Affiliations:** 1 Internal Medicine, Parkview Health, Fort Wayne, USA; 2 Internal Medicine, Azad Jammu Kashmir Medical College, Muzaffarabad, PAK; 3 Internal Medicine, Northeast Internal Medicine Associates, Lagrange, USA; 4 Gastroenterology, Parkview Health, Fort Wayne, USA; 5 Interventional Oncology and Surgical Endoscopy (IOSE), Parkview Health, Fort Wayne, USA

**Keywords:** bouveret's syndrome, duodenal bulb obstruction, gallstone extraction, gastric outlet obstruction, upper endoscopy

## Abstract

Bouveret syndrome is an uncommon condition that leads to gastric outlet obstruction. Diagnosis and treatment may get delayed due to non-specific presentation and rarity of the condition, which is associated with high morbidity and mortality. We present a case of a 62-year-old female who presented with epigastric pain and nausea and was diagnosed with Bouveret syndrome. Despite the low success rate, endoscopy should be the first-line diagnostic and therapeutic procedure and is associated with a low mortality rate as compared to surgical intervention. This case underscores the diagnostic and therapeutic challenges of Bouveret syndrome.

## Introduction

Mechanical gastric outlet obstruction caused by gallstones is called Bouveret syndrome. It is a rare cause of gallstone ileus (1-3%) [1-4] and gastric outlet obstruction [1,3]. The offending stone most commonly (about 60%) travels down from the biliary tree via a cholecystoduodenal fistula [1,5-7]. Clinical diagnosis could be delayed as the condition is rare [1,4]. This condition occurs most commonly in older females (in 70s) [1,2]. We discuss a rare case of gastric outlet obstruction in a 62-year-old female caused by a gallstone in the duodenal bulb. Duodenal bulb is a rare site of gallstone impaction as after a literature search most affected site is the terminal ileum (50-90%), followed by jejunum and proximal ileum (20-40%) [8]. Rigler’s triad, characterized by radiological findings of bowel obstruction, pneumobilia, and an ectopic gallstone, is a hallmark feature indicative of Bouveret syndrome [1,8].

This article was previously presented as a meeting abstract at the 2024 ACG Annual Scientific Meeting on October 29, 2024.

## Case presentation

**Figure 1:**
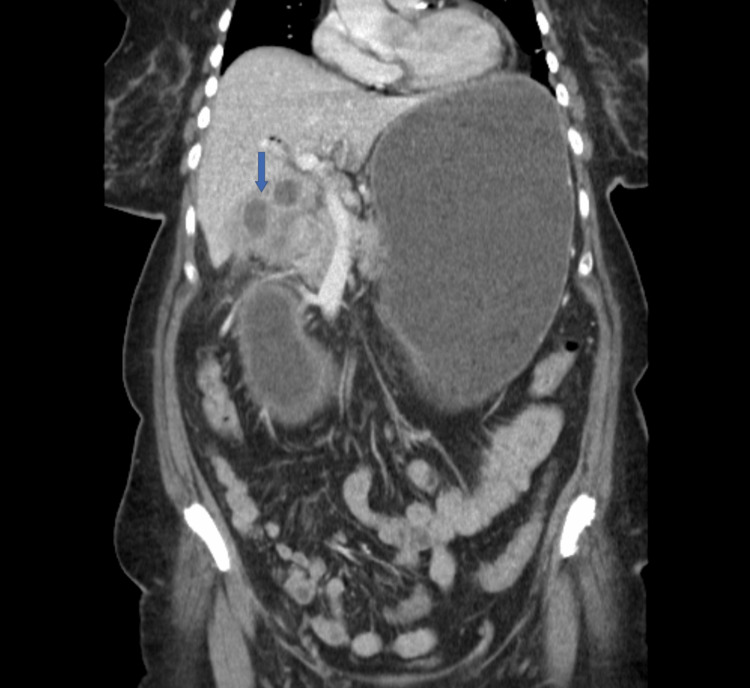
Cholecystoduodenal fistula (blue arrow) and obstruction causing gastric distention

**Figure 2:**
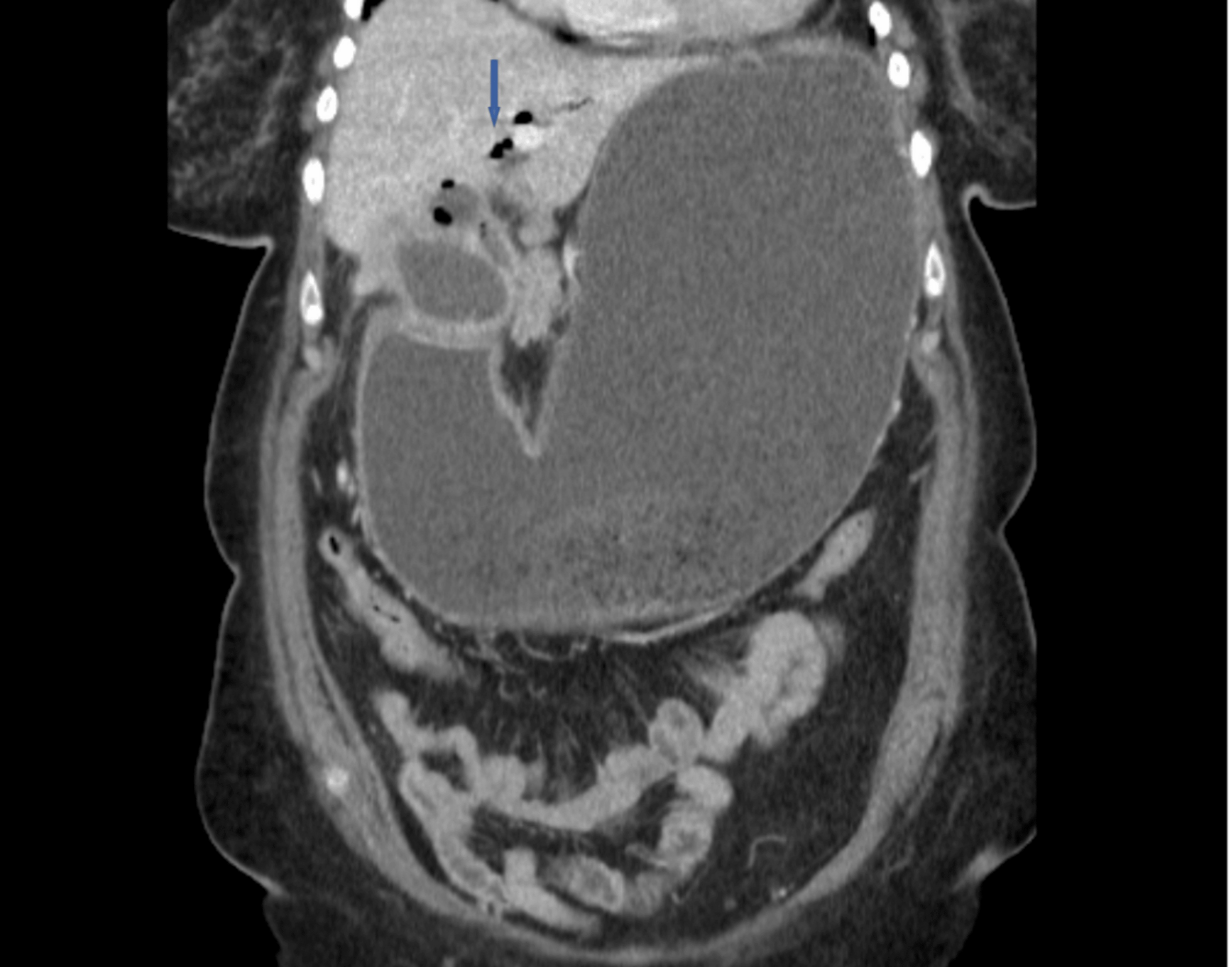
Biliary air in patient with gastric outlet obstruction due to a gallstone

A 62-year-old Caucasian woman, with a medical history of hypertension and diabetes, reported experiencing epigastric pain and nausea for the past month. One day before the presentation, the patient started vomiting and epigastric pain intensified. Examination revealed mild epigastric tenderness. Laboratory results showed normal complete blood count (CBC), basal metabolic panel (BMP), and unremarkable liver enzymes, including aspartate aminotransferase (AST), alanine aminotransferase (ALT), bilirubin, and alkaline phosphatase (ALP). A computed tomography (CT) scan of the abdomen and pelvis revealed a distended stomach filled with fluid and debris, along with signs of inflammation surrounding the pylorus and proximal small intestine, as well as the presence of air within the biliary tree. The remainder of the small bowel and colon were normal (Figures 1-2).

**Figure 3:**
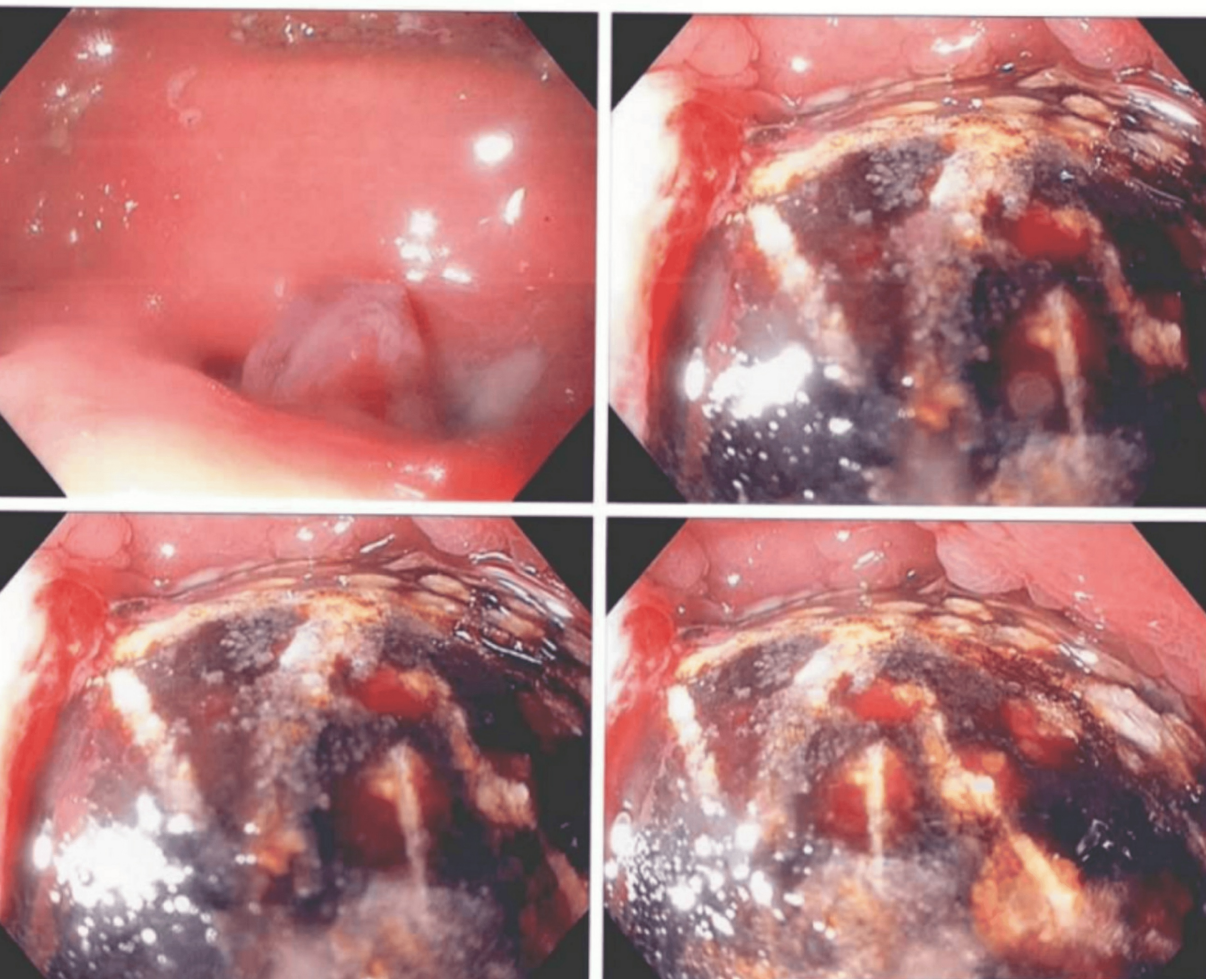
Gallstone on EGD EGD - esophagogastroduodenoscopy

An esophagogastroduodenoscopy (EGD) showed fluid and debris in the dilated stomach. An inflammatory polyp was seen near the pylorus. No abnormalities were found in the body, antrum, fundus, or cardia. A gallstone was identified as having eroded from the gallbladder into the duodenal bulb (Figure 3).

An attempt to retrieve the gallstone was unsuccessful because no device could be maneuvered around the impacted stone. A plan for an endoscopic electrohydraulic lithotripsy (EHL) was made. The known gallstone was identified in the duodenal bulb. Using EHL, attempts were made to destroy the stone. Multiple attempts using the through-the-scope (TTS) lithotripter were unsuccessful. Additional attempts were made using the Holmium laser. Despite multiple attempts and multiple techniques, the stone was unable to fully clear.

The following day, the patient underwent the surgical procedure (exploratory laparotomy, gastrotomy, removal of impacted gallstone, intraoperative duodenoscopy, repair of gastrotomy, Witzel jejunostomy), and post-op diagnosis of cholecystoduodenal fistula, impacted gallstone gastric was made.

The patient was kept under observation in the hospital and then discharged home on post-op day 12.

## Discussion

Bouveret syndrome is an uncommon condition that leads to obstruction of the gastric outlet [1-3]. Gallstone ileus is an extremely uncommon complication of cholelithiasis, occurring in only 0.3-0.5% of cases [4]. Risk factors for Bouveret syndrome include elderly females with a history of cholelithiasis or stones more than 2 cm [1]. Gallstone usually reaches the gastrointestinal tract via a fistula, either by pressure necrosis of the gallbladder wall or if the inflamed gallbladder is adherent to the gut [1]. Diagnosis can be challenging due to the nonspecific nature of the symptoms [1,3]. As this condition is present mostly in elderly patients and misdiagnosis could happen, mortality is between 12-20%. The most prevalent symptoms include nausea and vomiting (85%) and epigastric pain (70%) [1,3,7]. Less common presentations could be hematemesis [1,3] and/or melena [3]. The presence of Rigler’s triad, which includes imaging findings of obstruction, pneumobilia, and an ectopic gallstone, is considered pathognomonic [1]. A CT scan increases the accuracy of Rigler’s triad [1,3]. CT usually demonstrates pneumobilia or fistula and gallstone in approximately 50% of the cases [3].

Treatment options include endoscopic, laparoscopic, or open/surgical disimpaction of the stone [1]. An endoscopic approach is preferred [1-3]. The endoscopic approach (diagnostic and potentially therapeutic) is preferred due to the lowest morbidity and mortality and is now the first-line option [1,3,9]. Endoscopic options for disimpaction of stone in Bouveret syndrome include endoscopic baskets, mechanical, electrohydraulic, laser and extracorporeal shockwave lithotripsy (ESWL), and endoscopic intervention with surgical intervention [3,4]. Endoscopic disimpaction or retrieval of the stone with or without lithotripsy is the first-line intervention despite its low success rate (<10%) [1,7] due to the advanced age of the patients who present with Bouveret syndrome, and they usually have multiple comorbidities [4]. Endoscopy is limited in both the diagnosis (two-thirds of the stones are usually not visualized due to submucosal invasion) [1,3] and in successfully removing the stone [3].

If mechanical lithotripsy fails, EHL and laser lithotripsy are the options [3]. As, in Bouveret syndrome, stones are large, the maximum EHL intensity is usually required to fragment these stones [9]. Laser lithotripsy is superior to ESWL due to continuous endoscopic control during the procedure and higher efficacy [9]. Due to a very low success rate (4%), ESWL should be avoided [9].

A laparoscopic approach (if available) with gastrotomy or duodenotomy is preferred [3] over an open approach due to lower morbidity and mortality as compared to the open approach, although the failure rate approaches 50% with the laparoscopic approach [1]. Open surgical approach (enterolithotomy or gastrostomy) is the last resort due to high morbidity and mortality [1,3,9]. Endoscopy could be used with an open approach to mobilize the stone into a more favorable place to perform open surgery [1,3]. It is also recommended to assess the rest of the small intestine to exclude other stones to prevent distal obstruction [1]. Cholecystectomy with fistula repair during or after laparoscopic or open treatment is an area of debate [3,9]. Some studies recommend single-stage cholecystectomy or delayed cholecystectomy to prevent recurrence [1]. The one-stage approach offers the benefit of reducing the need for subsequent procedures while lowering the risk of complications, such as gallbladder inflammation (cholecystitis), recurrent gallstone ileus, bile duct infections (cholangitis), bile duct cancer (cholangiocarcinoma), and gallbladder cancer [7]. Others recommend against it as the mortality rate with simple stone extraction is 12% and goes up to 20-30% if combined with one-stage cholecystectomy [4]. Fistula closure is not generally recommended as spontaneous repair or closure has been documented in more than 50% of the cases [1]. Treatment options for Bouveret syndrome, whether surgical or non-surgical, should be individualized based on the patient's clinical presentation and underlying comorbidities [10].

## Conclusions

Bouveret syndrome is an uncommon condition that leads to obstruction of the gastric outlet. Diagnosis and treatment may get delayed due to non-specific presentation and rarity of the condition, which is associated with high morbidity and mortality. Endoscopy, despite its low success rate, should be the first-line diagnostic and therapeutic procedure and is associated with a low mortality rate as compared to surgical intervention. If the endoscopic approach fails to retrieve the stone, surgical options should be used.
